# A novel *POLE* mutation associated with cancers of colon, pancreas, ovaries and small intestine

**DOI:** 10.1007/s10689-015-9803-2

**Published:** 2015-04-10

**Authors:** Maren F. Hansen, Jostein Johansen, Inga Bjørnevoll, Anna E. Sylvander, Kristin S. Steinsbekk, Pål Sætrom, Arne K. Sandvik, Finn Drabløs, Wenche Sjursen

**Affiliations:** Department of Laboratory Medicine, Children’s and Women’s Health, Faculty of Medicine, Norwegian University of Science and Technology, 7491 Trondheim, Norway; Department of Pathology and Medical Genetics, St. Olavs Hospital, Trondheim University Hospital, 7006 Trondheim, Norway; Department of Cancer Research and Molecular Medicine, Faculty of Medicine, Norwegian University of Science and Technology, 7491 Trondheim, Norway; Department of Public Health and General Practice, Faculty of Medicine, Norwegian University of Science and Technology, 7491 Trondheim, Norway; Department of Computer and Information Science, Faculty of Information Technology, Mathematics and Electrical Engineering, Norwegian University of Science and Technology, 7491 Trondheim, Norway; Department of Gastroenterology, St. Olavs Hospital, Trondheim University Hospital, 7006 Trondheim, Norway; Centre of Molecular Inflammation Research, Norwegian University of Science and Technology, 7491 Trondheim, Norway

**Keywords:** Colorectal cancer, Polymerase epsilon, *POLE*, Germline mutation

## Abstract

In some families there is an increased risk for colorectal cancer, caused by heritable, but often unidentified genetic mutations predisposing to the disease. We have identified the likely genetic cause for disease predisposition in a large family with high burden of colorectal adenomas and carcinomas, in addition to extra-colonic cancers. This family had previously been tested for known cancer susceptibility genes, with negative results. Exome sequencing was used to identify a novel mutation, c.1373A>T (p.Tyr458Phe), in the gene for DNA polymerase epsilon catalytic subunit (*POLE*). This mutation is located in the active site of the exonuclease domain of the enzyme, and affects a residue that has previously been shown to be important for exonuclease activity. The first predisposing mutation identified in *POLE* (c.1270C>G, p.Leu424Val) was associated with colorectal cancer only, but another mutation with a broader tumour spectrum (c.1089C>A, p.Asn363Lys) has recently been reported. In the family described in the present study, carriers generally have multiple colorectal adenomas and cancer of colon, pancreas, ovaries and small intestine which represents an important broadening of the tumour spectrum of *POLE* mutation carriers. We also observe a large phenotypic variation among the *POLE* mutation carriers in this family, most likely explained by modifying variants in other genes. One *POLE* mutation carrier has a novel variant in *EXO1* (c.458C>T, p.Ala153Val), which may contribute to a more severe phenotype. The findings in this study will have important implications for risk assessment and surveillance of *POLE* mutation carriers.

**Electronic supplementary material:**

The online version of this article (doi:10.1007/s10689-015-9803-2) contains supplementary material, which is available to authorized users.

## Introduction

About one-third of all colorectal cancer (CRC) cases are presumed to be caused by hereditary factors [1–3]. The genetic basis for predisposition is unknown in the majority of familial CRC cases, and only about 5 % of all CRC cases are associated with CRC syndromes caused by highly penetrant mutations in known CRC predisposing genes [4]. Lynch Syndrome is the most commonly occurring CRC syndrome and is caused by a germline mutation in one of the DNA mismatch repair (MMR) genes *MLH1* (MIM *120436), *MSH2* (MIM *609309), *MSH6* (MIM *600678) or *PMS2* (MIM *600259). Polyposis is rare in Lynch Syndrome, but affected individuals develop colonic adenomas and carcinomas with higher frequency compared to the general population [5]. Familial adenomatous polyposis (FAP) is the second most common hereditary CRC syndrome and is caused by a germline mutation in the *APC* gene (MIM *611731). Classical FAP is characterized by hundreds to thousands of colonic adenomas starting to appear in adolescence. Attenuated FAP is a less severe form of the condition with fewer adenomas and later onset of disease [6]. MutYH-associated polyposis (MAP) is caused by biallelic mutations of the *MUTYH* gene (MIM *604933). Adenomatous polyps predominate in MAP but hyperplastic polyps are also common. Peutz–Jeghers Syndrome, Juvenile Polyposis Syndrome and Cowden Syndrome are conditions characterized by hamartomatous polyposis. Peutz–Jeghers Syndrome and Cowden Syndrome are caused by a mutation in *STK11* (MIM *602216) and *PTEN* (MIM +601728), respectively, whereas Juvenile Polyposis Syndrome is caused by mutations in either *BMPR1A* (MIM *601299) or *SMAD4* (MIM *600993). Recently a new CRC predisposing syndrome named polymerase proofreading-associated polyposis (PPAP) was described [7]. This syndrome is caused by germline mutations in *POLE* (MIM *174762) or *POLD1* (MIM *174761), encoding the catalytic and proofreading subunit of the DNA polymerase ε and δ enzyme complexes, respectively.

Currently, clinical presentation of CRC patients is used to guide genetic testing. Although there are some distinct clinical features associated with each CRC syndrome, the phenotypes overlap extensively and this can complicate phenotype-guided genetic testing and counselling. For several of the above mentioned syndromes, affected individuals can present with varying number of adenomas (typically 10–100) at a young age, which can develop into CRC if left untreated. The extra-colonic tumour spectrum may also be somewhat overlapping for several of the CRC syndromes involving endometrium, stomach, ovaries, pancreas, small bowel and brain [8].

In the present study we describe a large family with high burden of colorectal adenomas and carcinomas in addition to extra-colonic cancers. Initially, three separate families were identified, but they were later found to have shared ancestry. Genetic examinations of family members started in 1995 and since then several CRC predisposing genes have been analysed without identification of a causal mutation. Due to the striking dominant inheritance in this family, we strongly suspected a highly penetrant mutation as the cause of cancer predisposition. By exome sequencing we identified a novel mutation in *POLE* which seems to explain the cancer predisposition. Further, we discuss whether modifying effects of variants in other genes may explain the phenotypic variation observed among the *POLE* mutation carriers.

## Materials and methods

### Recruitment of participants

The power of family-based studies can be optimized by careful selection of candidates for sequencing. However, because of ethical and legal constraints, recruitment of individuals to this study had to be done through members of the family who had previously received genetic counselling based on their personal concern for developing cancer. We asked this initial group of family members to distribute our invitation letter to additional relatives. This broadened the possibility to recruit participants but gave no guarantee of reaching specific important individuals. Although we were able to recruit enough informative participants to identify a likely causal mutation for predisposition in this family, it is clear that too strict legal constraints for recruitment to family-based studies can hamper such efforts.

### Description of pedigree

**Fig. 1:**
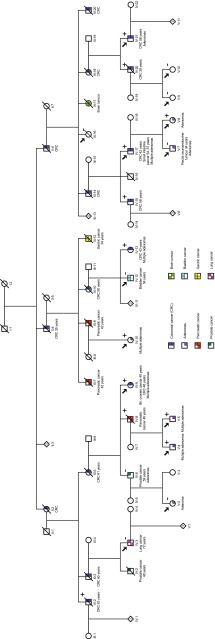
Pedigree of the family with the c.1373A>T (p.Tyr458Phe) mutation in *POLE*. Exome sequencing was performed on samples from the individuals indicated by an *arrow*. A *plus* (+) indicates the heterozygous mutation carriers and a *minus* (−) indicates the family members negative for the mutation. The pedigree has been modified to protect privacy of the family

All patient samples and clinical information was obtained with informed written consent and the study was approved by the Regional Committee for Medical and Health Research Ethics of Central Norway (approval 2012/1707). The studied family has been followed at St. Olavs Hospital, Norway, for two decades, and the pedigree includes more than 100 individuals. It consists of more than 10 second generation individuals and about 30 individuals each for third, fourth and fifth generation. About 40 individuals have been affected with cancer or adenomas. The majority of these aberrations were localized in the colon but also in pancreas, ovaries, urinary tract, stomach, small intestine, prostate and lung. To protect privacy, a modified pedigree is shown in Fig. 1. Initially, this family was believed to be affected with a polyposis syndrome because of their tendency to develop polyps. Their phenotype resembles a less severe form of polyposis like attenuated FAP or MAP. However, no germline mutation was detected in either *APC* or *MUTYH*. The family also fulfilled Amsterdam Criteria and Bethesda Guidelines presenting with CRC and/or other Lynch Syndrome associated cancers or adenomas in all generations, several below 50 years of age. The MMR genes (*MLH1*, *MSH2*, *MSH6* and *PMS2*) were tested for germline mutations, but no abnormalities could be detected in any of these genes. Patient IV:9 presented with bilateral ovary cancer at the age of 40, CRC at the age of 48 and multiple adenomas on subsequent annual controls. In addition to the above mentioned genes, the patient was tested for pathogenic alterations in *BRCA1* and *BRCA2*. No germline mutation could be detected in these genes either. Several of the affected individuals started developing adenomas in their late twenties (V:4, V:5, V:7 and V:8) with new adenomas detected and removed during every successive annual colonoscopy. The youngest patient underwent polypectomy at age 26 (V:4). Individual IV:17 had the first colonoscopy at age 35, finding multiple adenomas with mild to high-grade dysplasia. At age 36 he underwent left sided colectomy because of polyposis. Several adenomas were detected yearly in the remaining colon until the age of 42, when he was diagnosed with CRC and underwent colectomy with ileorectal anastomosis. On annual controls he continued to present with adenomas in rectum, small intestine and stomach. At age 54 and 57 the patient was diagnosed with cancer in jejunum and duodenum, respectively. Individual III:16 died at 89 years of age without any evidence of colorectal or other cancers. There are no malignancies in the descendants of this person (not shown in pedigree). We therefore assessed III:16 to be truly unaffected.

### Exome capture and sequencing

We exome sequenced DNA samples from 14 family members (III:16, IV:3, IV:9, IV:10, IV:12, IV:17, IV:21, V:2, V:4, V:5, V:7, V:8, V:9, V:10), both affected and unaffected, to identify the causative mutation predisposing to CRC in this family (see Fig. 1 and Online Resource 1 for overview of sequenced individuals). Exome capture was performed according to manufacturer’s protocol, using SureSelectXT Human All Exon V5+UTRs (Protocol version 1.6, Agilent Technologies, Santa Clara, CA). Briefly, the samples were quantified using Qubit^®^ 2.0 Fluorometer (Life Technologies, Carlsbad, CA). Genomic DNA was fragmented to approximately 170 bp by sonication using Covaris M220 Focused-ultrasonicator™ (Covaris, Woburn, MA). Fragment sizes were determined on Agilent 2100 Bioanalyzer (Agilent Technologies). Library concentrations were measured using Qubit^®^ 2.0 Fluorometer and StepOnePlus™ Real-Time PCR System (Life Technologies). The libraries were sequenced on Illumina HiSeq 2500 (Illumina, San Diego, CA) with 2 × 100 bp paired end sequencing.

### Data analysis, filtering and annotation

Exome sequencing data was aligned to the human genome (hg19, UCSC assembly, February 2009) using the Burrows–Wheeler–Aligner [9]. PCR duplicated sequences were removed with Picard-tools [10] and BAM files were converted with SAMtools [11]. Variant calling was done according to GATK Best Practices recommendations [12, 13] using GATK version 3.1, including local realignment around indels and recalibration of quality scores [14]. Quality control of called variants was performed using GATK VariantFiltration with parameter settings according to recommendations in SEQanswers exome sequencing analysis guide [15]. Variants were annotated with ANNOVAR [16]. Filtering was done using the filtering tool FILTUS version 0.99-9 [17]. We used two filtering strategies to find causative variant(s). The first approach was based on disease status which would enable us to find variants in potentially novel cancer predisposing genes. The second approach utilized a predefined CRC gene panel which would aid in finding predisposing variants in genes already known to be associated with CRC. The initial filtering steps were identical for the two approaches. These initial steps included removal of all variants that were synonymous, identified in 1000 Genomes Project with MAF <0.001, present in dbSNP build 138 and not flagged as “PASS” after quality control. In the first filtering approach, based on disease status, the remaining variants from 7 individuals (IV:9, IV:10, IV:17, V:4, V:5, V:7 and V:8) classified as “affected” based on their phenotypes were filtered against 1 individual (III:16) classified as “unaffected” (see Online Resource 1 for overview). The remaining individuals (V:2, IV:21, V:10, IV:3 and V:9) were not included in this filtering analysis because they could not be confidently classified as “affected” or “unaffected”. In the second filtering approach, all exome sequenced samples were included and we utilized a predefined panel consisting of genes previously known to be associated with CRC (Online Resource 2). Variants present in the unaffected individual (III:16) were filtered out. For patient V:7 we also applied a panel of genes (Online Resource 3) in which a mutation may predispose to formation of endocrine tumours. Alamut software (Interactive Biosoftware, Rouen, France) was utilized for further annotation of variants. The following tools and measures were used to assess the functional impact at protein level of observed variants: Grantham’s distance [18], PhyloP [19], SIFT [20], MutationTaster [21], PolyPhen2 [22] and MutationAssessor [23]. Cutoff values used by the respective prediction programs to determine functional impact of variants is given in Table 1. Multiple alignment of protein sequences was performed with Clustal Omega [24] and ESPript 3.0 [25]. Domains were annotated according to Shevelev and Hübscher [26]. Active site residues were annotated according to the Conserved Domains Database (CDD) [27]. Known variants were annotated according to data from COSMIC v71 [28], ExAC Version 0.2 [29] and dbSNP Build 142 [30].

**Table 1:** Variants identified by sequencing with potential functional impact

Gene	Change DNA/AA	G.dist^a^	PhyloP^b^	SIFT^c^	MutationTaster^d^	PolyPhen2^e^	SNPs3D^f^	MutationAssessor^g^	TCGA	Samples
*POLE* NM_006231.2	c.1373A>T p.Tyr458Phe	22	4.97	0.00	1	1.00	−1.92	3.86	0	**III:2**, **IV:8**, IV:9, IV:10, **IV:13**, **IV:15**, IV:17, **IV:20**, IV:21, V:4, V:5, V:8
*BMPR1A* NM_004329.2	c.1379T>C p.Met460Thr	81	3.35	0.00	1	0.49	0.14	1.81	1 ^h^	V:7
*EXO1* NM_003686.4	c.458C>T p.Ala153Val	64	6.18	0.02	1	0.99	−3.07	3.90	0	IV:17
*CHEK2* NM_007194.3	c.1100del p.Thr367Metfs*15	–	–	–	–	–	–	–	3^i^	V:2
*LAMB4* NM_007356.2	c.5265del p.Lys1755Asnfs*11	–	–	–	–	–	–	–	1^j^	IV:6, V:2

The table shows gene name, variant at DNA and protein level, prediction of functional impact (Grantham’s physiochemical distance between pairs of amino acids, PhyloP basewise conservation score, SIFT, MutationTaster, PolyPhen2, SNPs3D, MutationAssessor; see footnotes for explanation of score values), number of samples with this variant in The Cancer Genome Atlas and the individuals in which the DNA variant was found. Individuals that were added after the initial exome sequencing (only Sanger sequenced) are shown in bold

^a^Grantham’s distance from 5 to 215

^b^Sites predicted to be conserved are assigned positive scores, while sites predicted to be fast-evolving are assigned negative. Range −20 to +10 for the human genome

^c^Score values from 0 to 1. The amino acid substitution is predicted to be damaging if the score is ≤0.05, and tolerated if the score is >0.05

^d^Prediction of a disease-causing variant. *P* value close to 1 indicates a high confidence of the prediction

^e^Prediction of a change being damaging (>0.85), possibly damaging (0.15–0.85) or benign (<0.15) (HumVar)

^f^A positive score indicates a variant classified as non-deleterious, and a negative score indicates a deleterious variant. The larger the score, the more confident classification

^g^Uses functional impact score to predict non-functional <1.938 or >1.938 functional impact

^h^Found in colorectal adenocarcinoma as a somatic change

^i^Found in one invasive breast carcinoma as a germline variant with loss of heterozygozity in the tumour. Also found in two cell lines

^j^Found as a somatic change in one stomach adenocarcinoma from 63 years old male. Copy number status for the gene was diploid

All variants identified in the present study and reported here have been submitted to LOVD 3.0 shared installation (http://databases.lovd.nl/shared/genes).

### Confirmatory Sanger sequencing

DNA from EDTA-preserved whole blood or paraffin-embedded tissue was analysed to confirm the variants c.1373A>T (*POLE*), c.1739T>C (*BMPR1A*), c.458C>T (*EXO1*), c.1100del (*CHEK2*) and c.5265del (*LAMB4*) detected by exome sequencing, and to test additional family members for the respective variants. PCR was performed using AmpliTaq Gold^®^ 360 MasterMix and 360 GC Enhancer (Life Technologies). Cycle sequencing reaction was performed with BigDye^®^ Terminator v3.1 (Life Technologies) and subsequent capillary electrophoresis was performed by the 3130xl Genetic Analyzer (Life Technologies). Sanger sequencing data was analysed using SeqScape Software v2.5 (Life Technologies).

### Validation cohort

Sequencing data from 95 CRC patients fulfilling the Amsterdam criteria but without identified germline mutation (previously tested for *MLH1*, *PMS2*, *MSH6*, *MSH2*, *APC* and *MUTYH*) was investigated for the *POLE* mutation. The library was prepared according to the manufacturer’s instructions using a custom Haloplex kit (Agilent Technologies) and was subsequently sequenced on a HiSeq 2500 (Illumina) with 2 × 100 bp paired end sequencing.

## Results and discussion

### Filtering of variants

The family included in this study show an autosomal dominant inheritance pattern with colorectal adenomas, carcinomas and other extra-colonic cancers detected in every successive generation (Fig. 1). Several family members had previously been tested for mutations in *APC*, *MUTYH*, *MLH1*, *PMS2*, *MSH2*, *MSH6*, *BRCA1* and *BRCA2*, with negative results. We therefore exome sequenced samples from 14 family members, both affected and unaffected, to detect any cancer predisposing mutation in this family. Average coverage across all sample was 152× (see Online Resource 1 for average coverage and the percentage of target regions covered in each sample) and approximately 25,000 variants were initially detected in each individual. These variants were first filtered against the 1000 Genomes Project and dbSNP, in order to focus on rare variants. Further, all synonymous variants and variants that did not pass quality filters were removed. This reduced the list to approximately 200 variants for each individual. Because of the broad spectrum of cancers and the varying phenotypes in this family, we used two complementary strategies for further variant filtering. The filtering strategy based on disease status identified 4 variants shared by the 7 affected individuals, none of which were assessed to be likely causative in terms of gene function or functional impact of the variants. The number of variants increased to 8, 15, 24, 42 and 105 if the number of affected individuals was reduced to at least 6, 5, 4, 3 or 2, respectively. This corresponds to assuming that at least one individual may have developed CRC by an alternative pathway, which is not unreasonable in a large family (>100 individuals). The gene panel strategy resulted in 8 variants in 8 different genes. Of these, 1 variant was shared by 6 affected individuals, 1 variant was present in two affected individuals and the remaining 6 variants were private. A novel mutation, c.1373A>T (p.Tyr458Phe), in *POLE* (NM_006231.2), present in 6 of the 7 patients classified as “affected” (V:4, V:5, V:8, IV:9, IV:10 and IV:17) and not present in the “unaffected” individual (III:16) was assessed to be the most likely causative mutation. This mutation was identified by both filtering methods. The *POLE* mutation was subsequently also found in 6 additional affected individuals (III:2, IV:8, IV:13, IV:15, IV:20 and IV:21). Samples from III:2, IV:8, IV:13, IV:15 and IV:20 were not available for exome sequencing, but were sequenced by the Sanger method. Individual IV:21 was exome sequenced, but could not be confidently classified as “affected” prior to filtering as only one adenoma had been detected in this patient, and the latest performed colonoscopy was 8 years ago. However, colonoscopy performed after exome sequencing of IV:21 revealed CRC and several adenomas. The individual classified as “affected” without *POLE* mutation (V:7) was found to harbour a novel variant in *BMPR1A*. See Fig. 1 and Online Resource 1 for overview of *POLE* mutation carriers. Only variants that are likely to have functional impact at the protein level and relevance to cancer predisposition are presented here (Table 1, see further discussion below). All these variants have been confirmed by Sanger sequencing.

### The *POLE* mutation c.1373A>T (p.Tyr458Phe)

**Fig. 2:**
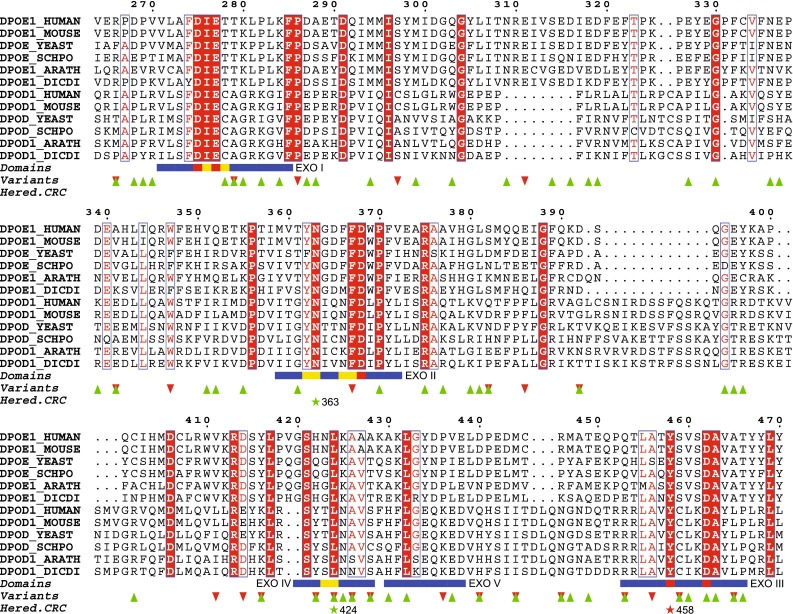
Multiple alignment of orthologous Polε and Polδ amino acid sequences. The alignment shows conserved positions in *blue boxes* (*boxes* with *red* background indicate completely conserved positions). The exonuclease domains (I–V) are indicated by *horizontal blue lines*. Essential residues of the DEDDy subfamily are indicated by *yellow* (active site residues) and *red* (catalytic residues) *squares* within the exonuclease domains. Known variants according to COSMIC and ExAC/dbSNP are indicated with *red* (*filled triangle*) and *green* (*filled inverted triangle*) *triangles*, respectively. The positions of the previously identified pathogenic germline mutations in CRC, p.Leu424Val and p.Asn363Lys, are indicated by *green stars* and sequence positions. The position of the variant identified in this study, p.Tyr458Phe, is indicated by a *red star*

DNA polymerase ε catalytic subunit (Polε) is a large polymerase for leading-strand synthesis during DNA replication in eukaryotes (2286 aa; NP_006222.2), whereas DNA polymerase δ (Polδ) most likely is responsible for replication of the lagging strand [31]. The Polε enzyme contains both a polymerase domain and a 3′-5′-exonuclease domain, which contributes to a very high fidelity of replication. Pathogenic germline mutations in *POLE* or *POLD1* have recently been described to cause the CRC syndrome PPAP. This is a highly penetrant, autosomal dominant syndrome predisposing to development of multiple adenomas and carcinomas. Most of the previously reported pathogenic germline mutations in *POLE* and *POLD1* cluster around the active site of the exonuclease domain and impair exonuclease activity [7, 32–34], apparently without affecting polymerase activity. The catalytic subunit of Polε contains a DEDDy 3′-5′ exonuclease domain, and the name of this superfamily is from four completely conserved amino acids (DEDD) of the active site found in three sequence motifs (Exo I–III), with a specific Y-X(3)-D pattern at Exo III. An alignment indicating domains and active site residues of the DEDDy subfamily is shown in Fig. 2. The missense substitution p.Tyr458Phe identified in the present study is located in the active site Exo III motif of the exonuclease domain. All applied tools for predicting variant effects at the amino acid level predicted this mutation to have functional impact (Table 1). The tyrosine corresponds to the “y” in DEDDy and is completely conserved between species. This position is important for the exonuclease activity [35, 36], which has been shown to be significantly reduced in orthologues where the equivalent position has been mutated to phenylalanine, alanine or histidine (residues p.Tyr320 in Bacteriophage T4 DNA polymerase, p.Tyr497 in *E. coli* DNA pol I Klenow fragment, p.Tyr165 in ϕ29 DNA polymerase, p.Tyr577 in herpes simplex virus DNA polymerase) [37–40]. This will reduce the fidelity of DNA replication [39], leading to increased mutation rate [40]. The exact function of the conserved tyrosine is still unclear. The first step of the exonuclease reaction is formation of a hydroxide ion to attack the phosphodiester bond at the site of cleavage [35, 41]. Structural data of *E. coli* DNA pol I Klenow fragment indicate that the phenolic side chain of the conserved tyrosine residue orients the attacking hydroxide ion during transition state. Further, crystallographic structure of the Klenow fragment has shown that the tyrosine is hydrogen-bonded to the phosphate of the bond to be cleaved in the 3′-5′ exonuclease reaction [42]. Another study observed increased binding of DNA substrate to the exonuclease active site when the tyrosine was substituted with alanine [43]. These findings suggest that the conserved tyrosine is important for reorienting the DNA substrate from the binding conformation to the catalytically active conformation, making the DNA more accessible for hydrolysis [41, 43].

95 additional samples from CRC patients fulfilling the Amsterdam criteria but without identified germline mutation (previously tested for *MLH1*, *PMS2*, *MSH6*, *MSH2*, *APC* and *MUTYH*) were analysed for the *POLE* mutation encoding the p.Tyr458Phe alteration. One index patient diagnosed with CRC at 44 years of age, and cancer duodenum at 59 years of age, was found to harbour the *POLE* mutation. His deceased brother got CRC at 42 years of age, and his son has removed several adenomas at age 34. Analysis of archived paraffin-embedded tissue material from the brother and DNA from wole blood from his son revealed that both of them carried the *POLE* mutation. The parents of the index patient died in their early fifties and sixties, however, no material from them were available for testing. We could not find common ancestors in the two families described here, although this cannot be completely ruled out. Consequently, we have identified two apparently unrelated families with history of CRC with the same *POLE* mutation.

Data from The Cancer Genome Atlas (TCGA), The International Cancer Genome Consortium (ICGC) cancer genome projects and other cancer genomics studies was accessed through the Catalogue of Somatic Mutations in Cancer (COSMIC) [28] and cBioPortal for Cancer Genomics [44] to find somatic *POLE* alterations encoding a change at position p.Tyr458, with negative results (Table 1). The codon next to p.Tyr458 is identified as a mutational hotspot with p.Ser459Phe found in 4 different hyper-mutated CRCs without microsatellite instability [44–46]. Another study of samples from microsatellite stable CRCs identified the somatic mutations p.Ser459Phe and p.Thr457Met in two cases each [47]. The exonuclease domain was also checked against Exome Aggregation Consortium (ExAC) [29]. As shown in Fig. 2, no previously known somatic or germline mutations in p.Tyr458 were found.

The family members with the *POLE* mutation were all heterozygous for the mutation. Second hit by somatic mutation or loss of heterozygosity (LOH) was not investigated in this study. However, Palles et al. [7] tested for second hits by LOH in 39 tumours from 11 carriers of the *POLE* mutation encoding p.Leu424Val, and detected LOH in 10 of these tumours. Rohlin et al. [32] searched for second hit by somatic mutations or LOH of the wt allele in two tumours from two carriers of the *POLE* mutation encoding p.Asn363Lys, but no aberrations were identified. Results from these two studies indicate that a second hit might not be required, and that the increased error rate during replication from only one faulty copy of the *POLE* gene might be enough to drive cancer development in humans. However, model studies in mice reveal that although mutation frequency is increased in mice that are heterozygous for a *POLE* mutation, only the homozygous mice showed increased susceptibility to cancer [48]. This indicates that additional factors may be important. Whether *POLE* acts as a classic tumour suppressor gene is still unclear and further research is needed to clarify this.

### The tumour spectrum of *POLE* mutation carriers

The tumour spectrum of the patients with previously reported pathogenic mutations affecting the exonuclease domain of Polε differs substantially. Palles et al. [7] first reported a family with a *POLE* mutation encoding the p.Leu424Val alteration that was solely affected with colorectal carcinomas and adenomas, while *POLD1* mutation carriers, in addition to CRC, were affected with endometrial cancers. Rohlin et al. [32] recently described a family with a *POLE* mutation encoding the p.Asn363Lys mutation that had a broader tumour spectrum, including cancer in colon, endometrium, ovaries, brain and one single case of late onset pancreatic cancer. Spier et al. [49] reports several *POLE* mutation carriers with duodenal adenomas and one case of duodenal cancer. The present family seems to be predisposed to adenomas and carcinomas not only in colon and rectum, but also in the pancreas, small intestine, stomach, and ovaries. There are three cases of early onset pancreatic cancer in this family. The first (IV:8) was found to have the *POLE* mutation encoding p.Tyr458Phe, the second (III:9) was indirectly found to harbour the mutation through genetic testing of his child, while the third (III:7) was unavailable for testing and has no descendants. All three developed pancreatic cancer in their forties which is a considerably younger age of onset than average (~70 years) [50, 51]. Individual IV:17 had, in addition to CRC, two cancers in the small intestine. This strongly suggests that cancer of pancreas and small intestine is a part of the PPAP tumour spectrum. As suggested by Rohlin et al. [32] there might be a genotype-to-phenotype correlation for this gene, relating to the effect the amino acid substitution has on the protein. However, the discrepancy in tumour spectrum may also be explained by the sizes of the families. The family with p.Leu424Val mutation [7] is smaller with fewer affected individuals than the other two families, [10 and the present study]. Since CRC is the predominant effect of *POLE* exonuclease mutations, the limited tumour spectrum of that family may have occurred by chance. Two of the family members in the present study (IV:12 and IV:3) were affected with cancer in the urinary bladder at age 54 and lung cancer at age 70, respectively. However, they did not harbour the pathogenic *POLE* mutation. The lung cancer was most likely caused by environmental factors related to the person’s workplace.

### Carriers of the same *POLE* mutation have differing phenotypes

The *POLE* mutation carriers of the present family had differing phenotypes, most likely explained by modifying variants in other genes. Most of the p.Tyr458Phe carriers had a multiple-adenoma phenotype similar to MAP and attenuated FAP, while some had fewer adenomas or cancer of ovaries or pancreas more resembling Lynch Syndrome. Phenotypic variation among family members carrying the same *POLE* mutation is also observed in another study [7]. In the present study, the *POLE* mutation carrier with the most severe phenotype (IV:17) was also found to harbour the novel variant c.458C>T (p.Ala153Val) in *EXO1* (NM_003686.4) with predicted functional impact (Table 1). This variant was identified using the CRC genepanel strategy. Another SNP (rs143955774, c.458C>G, p.Ala153Gly) without reported frequency is located at the same position. *EXO1* encodes the enzyme Exonuclease 1 which belongs to the RAD2/XPG family of endo- and exonucleases. It exhibits 5′-3′-exonuclease and 5′-flap endonuclease activity and is involved in DNA repair, recombination, replication, and telomere integrity (reviewed in [52]). The residue p.Ala153 is located in the highly conserved XPG_2 site (PS00842), which includes a conserved pentapeptide, E-A-[DE]-**A**-[QS] (the residue in bold corresponds to p.Ala153), and is located next to one of the acidic residues of the active site involved in the catalytic mechanism of nuclease activity [53]. Studies of *POLE* mutant *Saccharomyces cerevisiae* strains deleted for *EXO1* show a markedly increased mutator phenotype compared to either of the single mutant strains [54–56]. This suggests that Exonuclease 1 is involved in correcting mismatches created by Polε during replication. Consequently, we postulate that the *POLE* and *EXO1* variants detected in DNA from patient IV:17 may have a combined effect leading to an increased mutation rate causing the even more severe phenotype observed in this patient. Identification of modifying loci causing discrepancy in the phenotypes of *POLE* mutations carriers obviously needs further research. It might be useful to investigate additional variants in genes coding for proteins involved in the same pathways as Polε, or look for variation in regulatory regions. It is also possible that common variants can have a modifying effect when combined with a pathogenic *POLE* mutation. Differences in phenotypes due to genetic modifiers have also been observed in Lynch Syndrome [57].

### Phenocopies may be explained by additional variants

Using the CRC gene panel filtering strategy we also identified other variants with potential functional impact (Table 1) in three family members without the *POLE* mutation. Initially these patients seemed to phenocopy *POLE* mutation carriers to some extent, but there were also clear differences.

Individual V:7, who was classified as “affected” but did not carry the *POLE* mutation, was found to have the mutation c.1379T>C (p.Met460Thr) in *BMPR1A* (NM_004329.2). He was initially thought to have a phenotype similar to his sister (V:8), who was found to carry the pathogenic *POLE* mutation, with hyperplastic polyps and adenomas from their twenties. Individual V:7 had previously only one tubular adenoma and one hyperplastic polyp detected, and during this project he developed a rectal neuroendocrine tumour. This type of tumour is not observed for any of the other family members, suggesting that this patient is affected with something other than PPAP. The *BMPR1A* variant has previously been found as a somatic change in a CRC analysed by the TCGA project, but prediction tools were inconsistent regarding functional impact (Table 1). In addition, considering that the *BMPR1A* variant was inherited from this person’s healthy mother (IV:18), who has not been examined with colonoscopy, and as neuroendocrine tumours are not associated with Juvenile Polyposis Syndrome, the variant was evaluated to be of uncertain clinical significance at this stage. A panel of genes related to endocrine tumours was also applied to the exome data of this individual, with negative results.

Individual V:2, who had a single adenoma detected at age 42, had the variants c.1100del in *CHEK2* (NM_007194.3), and c.5265del in *LAMB4* (NM_007356.2). The *CHEK2* variant is a well-known, low penetrant founder mutation mainly associated with breast cancer, but also CRC and prostate cancer [58–63]. A germline *LAMB4* variant has recently been reported in another CRC patient with somatic loss of the wild-type allele in the tumour [34]. *LAMB4* was consequently implicated to be a possible tumour-suppressor gene where mutations may predispose to CRC. Both the *CHEK2* and *LAMB4* variant were present in TCGA data, but the *CHEK2* variant as a germline mutation (Table 1). In the present study, the *LAMB4* variant, but not the *CHEK2* variant, was also found in the person’s father (IV:6) who was affected with prostate cancer at age 54 and two colorectal adenomas at age 60 and 67.

Since CRC is one of the most common malignancies in Norway it is likely that a large family like this also will have sporadic, non-hereditary cases of colorectal adenomas and cancer. There may also be additional genetic factors leading to a small increase in cancer susceptibility, like c.1100del in CHEK2, which together with environmental factors may lead to formation of adenomas or CRC. This clearly demonstrates the challenge of using phenotype-guided genetic testing combined with Sanger sequencing of single genes to find the genetic predisposition in familial CRC. Exome sequencing has successfully been applied to find the genetic cause for a wide range of Mendelian disorders (reviewed in [64]), but only a few studies have interrogated familial CRC [7, 32, 34, 65, 66]. It has previously been discussed that phenocopies and incomplete penetrance might hamper analysis of exome sequencing data when studying familial CRC [65]. In the current study we show that it is possible to identify the mutation causing the main burden of CRC in a family with multiple affected family members by using both “affected” and “unaffected” individuals, even in the presence of phenocopies. This clearly demonstrates the power of exome sequencing in genetic diagnostics of hereditary predisposition to cancer, and we anticipate that future studies will bring new insight in the molecular genetics of still unexplained cases of familial CRC.

## Conclusion

Exome sequencing of members of a family with high burden of colorectal adenomas and carcinomas, in addition to extra-colonic cancers, has identified the novel mutation c.1373A>T (p.Tyr458Phe) in *POLE* as a likely predisposing mutation. Previous functional and structural studies have shown that the position p.Tyr458 in Polε is important for exonuclease activity, and that the tumorigenic effect of p.Tyr458Phe is increased mutation rate due to reduced exonuclease activity, and consequently also reduced replication fidelity. The role of *POLE* in predisposition to cancer is consistent with previous studies where other mutations affecting the Polε exonuclease domain have been associated with CRC. Including the present study, *POLE* mutations have been associated with lesions in colon, rectum, small intestine, stomach, ovaries, endometrium, pancreas and brain. The overall evidence clearly suggests that extra-colonic cancers need to be taken into consideration in risk management and follow up of patients with *POLE* mutation. The varying phenotypes among *POLE* carriers are likely to be caused by modifying effects of other alleles, and further studies are necessary to provide personalized risk assessment. PPAP is a fairly recently described cancer susceptibility syndrome and guidelines regarding management of *POLE* and *POLD1* mutation carriers do not yet exist. It is important for this group of patients that such guidelines are implemented, incorporating the new knowledge on *POLE* mutations.

## Electronic supplementary material

Supplementary material 1 (PDF 47 kb)

Supplementary material 2 (PDF 41 kb)

Supplementary material 3 (PDF 71 kb)

## References

[1] Lichtenstein P, Holm NV, Verkasalo PK (2000). Environmental and heritable factors in the causation of cancer—analyses of cohorts of twins from Sweden, Denmark, and Finland. N Engl J Med.

[2] Johns LE, Houlston RS (2001). A systematic review and meta-analysis of familial colorectal cancer risk. Am J Gastroenterol.

[3] Grady WM (2003). Genetic testing for high-risk colon cancer patients. Gastroenterology.

[4] Jasperson KW, Tuohy TM, Neklason DW, Burt RW (2010). Hereditary and familial colon cancer. Gastroenterology.

[5] Lynch HT, Lynch PM, Lanspa SJ (2009). Review of the Lynch syndrome: history, molecular genetics, screening, differential diagnosis, and medicolegal ramifications. Clin Genet.

[6] Gala M, Chung DC (2011). Hereditary colon cancer syndromes. Semin Oncol.

[7] Palles C, Cazier J-B, Howarth KM (2013). Germline mutations affecting the proofreading domains of POLE and POLD1 predispose to colorectal adenomas and carcinomas. Nat Genet.

[8] Patel SG, Ahnen DJ (2012). Familial colon cancer syndromes: an update of a rapidly evolving field. Curr Gastroenterol Rep.

[9] Li H, Durbin R (2009). Fast and accurate short read alignment with Burrows–Wheeler transform. Bioinformatics.

[10] Broad Institute Picard Tools. http://broadinstitute.github.io/picard/. Accessed 22 Jan 2015

[11] Li H, Handsaker B, Wysoker A (2009). The sequence alignment/map format and SAMtools. Bioinformatics.

[12] DePristo MA, Banks E, Poplin R (2011). A framework for variation discovery and genotyping using next-generation DNA sequencing data. Nat Genet.

[13] Van der Auwera GA, Carneiro MO, Hartl C, Bateman A, Pearson WR, Stein LD (2013). From FastQ data to high-confidence variant calls: the genome analysis toolkit best practices pipeline. Current protocols in bioinformatics.

[14] McKenna A, Hanna M, Banks E (2010). The Genome Analysis Toolkit: a MapReduce framework for analyzing next-generation DNA sequencing data. Genome Res.

[15] How-to/exome analysis—SEQwiki. http://seqanswers.com/wiki/How-to/exome_analysis. Accessed 22 Jan 2015

[16] Wang K, Li M, Hakonarson H (2010). ANNOVAR: functional annotation of genetic variants from high-throughput sequencing data. Nucleic Acids Res.

[17] Vigeland MD Filtus. http://folk.uio.no/magnusv/filtus.html. Accessed 22 Jan 2015

[18] Grantham R (1974). Amino acid difference formula to help explain protein evolution. Science.

[19] Pollard KS, Hubisz MJ, Rosenbloom KR, Siepel A (2010). Detection of nonneutral substitution rates on mammalian phylogenies. Genome Res.

[20] Kumar P, Henikoff S, Ng PC (2009). Predicting the effects of coding non-synonymous variants on protein function using the SIFT algorithm. Nat Protoc.

[21] Schwarz JM, Cooper DN, Schuelke M, Seelow D (2014). MutationTaster2: mutation prediction for the deep-sequencing age. Nat Methods.

[22] Adzhubei IA, Schmidt S, Peshkin L (2010). A method and server for predicting damaging missense mutations. Nat Methods.

[23] Reva B, Antipin Y, Sander C (2011). Predicting the functional impact of protein mutations: application to cancer genomics. Nucleic Acids Res.

[24] Sievers F, Wilm A, Dineen D (2011). Fast, scalable generation of high-quality protein multiple sequence alignments using Clustal Omega. Mol Syst Biol.

[25] Robert X, Gouet P (2014). Deciphering key features in protein structures with the new ENDscript server. Nucleic Acids Res.

[26] Shevelev IV, Hübscher U (2002). The 3′ 5′ exonucleases. Nat Rev Mol Cell Biol.

[27] Marchler-Bauer A, Zheng C, Chitsaz F (2013). CDD: conserved domains and protein three-dimensional structure. Nucleic Acids Res.

[28] Forbes SA, Beare D, Gunasekaran P (2014). COSMIC: exploring the world’s knowledge of somatic mutations in human cancer. Nucleic Acids Res.

[29] Cambridge M Exome Aggregation Consortium (ExAC). http://exac.broadinstitute.org/. Accessed 1 Dec 2014

[30] Sherry ST, Ward MH, Kholodov M (2001). dbSNP: the NCBI database of genetic variation. Nucleic Acids Res.

[31] Hogg M, Osterman P, Bylund GO (2014). Structural basis for processive DNA synthesis by yeast DNA polymerase ɛ. Nat Struct Mol Biol.

[32] Rohlin A, Zagoras T, Nilsson S (2014). A mutation in POLE predisposing to a multi-tumour phenotype. Int J Oncol.

[33] Valle L, Hernández-Illán E, Bellido F (2014). New insights into POLE and POLD1 germline mutations in familial colorectal cancer and polyposis. Hum Mol Genet.

[34] Smith CG, Naven M, Harris R (2013). Exome resequencing identifies potential tumor-suppressor genes that predispose to colorectal cancer. Hum Mutat.

[35] Beese LS, Steitz TA (1991). Structural basis for the 3′-5′ exonuclease activity of *Escherichia coli* DNA polymerase I: a two metal ion mechanism. EMBO J.

[36] Brautigam CA, Steitz TA (1998). Structural principles for the inhibition of the 3′-5′ exonuclease activity of *Escherichia coli* DNA polymerase I by phosphorothioates. J Mol Biol.

[37] Derbyshire V, Grindley ND, Joyce CM (1991). The 3′-5′ exonuclease of DNA polymerase I of *Escherichia coli*: contribution of each amino acid at the active site to the reaction. EMBO J.

[38] Abdus Sattar AK, Lin TC, Jones C, Konigsberg WH (1996). Functional consequences and exonuclease kinetic parameters of point mutations in bacteriophage T4 DNA polymerase. Biochemistry.

[39] Soengas MS, Esteban JA, Lázaro JM (1992). Site-directed mutagenesis at the Exo III motif of phi 29 DNA polymerase; overlapping structural domains for the 3′-5′ exonuclease and strand-displacement activities. EMBO J.

[40] Hwang YT, Liu BY, Coen DM, Hwang CB (1997). Effects of mutations in the Exo III motif of the herpes simplex virus DNA polymerase gene on enzyme activities, viral replication, and replication fidelity. J Virol.

[41] Elisseeva E, Mandal SS, Reha-Krantz LJ (1999). Mutational and pH studies of the 3′→5′ exonuclease activity of bacteriophage T4 DNA polymerase. J Biol Chem.

[42] Freemont PS, Friedman JM, Beese LS (1988). Cocrystal structure of an editing complex of Klenow fragment with DNA. Proc Natl Acad Sci USA.

[43] Lam WC, Van der Schans EJ, Joyce CM, Millar DP (1998). Effects of mutations on the partitioning of DNA substrates between the polymerase and 3′-5′ exonuclease sites of DNA polymerase I (Klenow fragment). Biochemistry.

[44] Cerami E, Gao J, Dogrusoz U (2012). The cBio cancer genomics portal: an open platform for exploring multidimensional cancer genomics data. Cancer Discov.

[45] Gao J, Aksoy BA, Dogrusoz U (2013). Integrative analysis of complex cancer genomics and clinical profiles using the cBioPortal. Sci Signal.

[46] The Cancer Genome Atlas Network (2012). Comprehensive molecular characterization of human colon and rectal cancer. Nature.

[47] Stenzinger A, Pfarr N, Endris V (2014). Mutations in POLE and survival of colorectal cancer patients—link to disease stage and treatment. Cancer Med.

[48] Albertson TM, Ogawa M, Bugni JM (2009). DNA polymerase epsilon and delta proofreading suppress discrete mutator and cancer phenotypes in mice. Proc Natl Acad Sci USA.

[49] Spier I, Holzapfel S, Altmüller J (2014). Frequency and phenotypic spectrum of germline mutations in POLE and seven other polymerase genes in 266 patients with colorectal adenomas and carcinomas. Int J Cancer.

[50] Tingstedt B, Weitkämper C, Andersson R (2011). Early onset pancreatic cancer—comparison against matched controls. Ann Gastroenterol.

[51] Ferrone CR, Brennan MF, Gonen M (2008). Pancreatic adenocarcinoma: the actual 5-year survivors. J Gastrointest Surg.

[52] Tran PT, Erdeniz N, Symington LS, Liskay RM (2004). EXO1-A multi-tasking eukaryotic nuclease. DNA Repair (Amst).

[53] Shen B, Nolan JP, Sklar LA, Park MS (1997). Functional analysis of point mutations in human flap endonuclease-1 active site. Nucleic Acids Res.

[54] Liberti SE, Larrea AA, Kunkel TA (2013). Exonuclease 1 preferentially repairs mismatches generated by DNA polymerase α. DNA Repair (Amst).

[55] Hombauer H, Campbell CS, Smith CE (2011). Visualization of eukaryotic DNA mismatch repair reveals distinct recognition and repair intermediates. Cell.

[56] Tran HT, Gordenin DA, Resnick MA (1999). The 3′→5′ exonucleases of DNA polymerases delta and epsilon and the 5′→3′ exonuclease Exo1 have major roles in postreplication mutation avoidance in Saccharomyces cerevisiae. Mol Cell Biol.

[57] Talseth-Palmer BA, Wijnen JT, Brenne IS (2013). Combined analysis of three Lynch syndrome cohorts confirms the modifying effects of 8q23.3 and 11q23.1 in MLH1 mutation carriers. Int J Cancer.

[58] Cybulski C (2004). A novel founder CHEK2 mutation is associated with increased prostate cancer risk. Cancer Res.

[59] Dong X, Wang L, Taniguchi K (2003). Mutations in CHEK2 associated with prostate cancer risk. Am J Hum Genet.

[60] Gronwald J, Cybulski C, Piesiak W (2009). Cancer risks in first-degree relatives of CHEK2 mutation carriers: effects of mutation type and cancer site in proband. Br J Cancer.

[61] Huijts PEA, Hollestelle A, Balliu B (2014). CHEK2* 1100delC homozygosity in the Netherlands—prevalence and risk of breast and lung cancer. Eur J Hum Genet.

[62] Meijers-Heijboer H, Wijnen J, Vasen H (2003). The CHEK2 1100delC mutation identifies families with a hereditary breast and colorectal cancer phenotype. Am J Hum Genet.

[63] Wasielewski M, Vasen H, Wijnen J (2008). CHEK2 1100delC is a susceptibility allele for HNPCC-related colorectal cancer. Clin Cancer Res.

[64] Rabbani B, Tekin M, Mahdieh N (2014). The promise of whole-exome sequencing in medical genetics. J Hum Genet.

[65] DeRycke MS, Gunawardena SR, Middha S (2013). Identification of novel variants in colorectal cancer families by high-throughput exome sequencing. Cancer Epidemiol Biomarkers Prev.

[66] Gylfe AE, Katainen R, Kondelin J (2013). Eleven candidate susceptibility genes for common familial colorectal cancer. PLoS Genet.

