# Supplementary material for: A novel POLE mutation associated with cancers of colon, pancreas, ovaries and small intestine
Source: Fam Cancer. 2015 Apr 10;14(3):437–48. doi: 10.1007/s10689-015-9803-2 (PMC4559173; doi:10.1007/s10689-015-9803-2)
Supplement: Supplementary file 1 — Supplementary material 1 (PDF 47 kb) [file 10689_2015_9803_MOESM1_ESM.pdf]

Online Resource 2: List of genes included in the CRC gene panel

Journal: Familial Cancer

Title: A Novel POLE Mutation Associated with Cancers of Colon, Pancreas, Ovaries and Small Intestine

Authors: Maren F. Hansen, Jostein Johansen, Inga Bjørnevoll, Anna E. Sylvander, Kristin S. Steinsbekk, Pål Sætrom, Arne K. Sandvik, Finn Drabløs, Wenche Sjursen.

Corresponding author:

Maren F. Hansen

Department of Laboratory Medicine, Children´s and Women´s Health, Faculty of Medicine, Norwegian University of Science and Technology, 7491 Trondheim, Norway.

Department of Pathology and Medical Genetics, St. Olavs Hospital, Trondheim University Hospital, 7006 Trondheim, Norway

maren.hansen@ntnu.no or maren.hansen@stolav.no

ACVRL1

AKR1C4

AKT1

APC

ATM

AURKA

AXIN1

AXIN2

BAX

BCLAF1

BGLAP

BLM

BMP2

BMP4

BMPR1A

BRCA1

BRCA2

BUB1

BUB1B

BUB3

CCDC18

CCND1

CCND2

CDH1

CDKN1A

CENPE

CHEK2

CTNNB1

DCC

DCLRE1A

DSG4

DUSP10

DUSP4

EIF3C

EIF3H

ENG

EPCAM

EPHB2

EXO1

FAM166A

FANCD2

FANCM

FLCN

GALNT12

GREM1

HAQ1

HELQ

KIF23

KIT

KLLN

LAMA3

LAMA5

LAMB4

LAMC1

LAMC3

LUC7L

MAML3

MCC

MLH1

MLH3

MRPL3

MSH2

MSH3

MSH6

MUTYH

MYC

MYH11

NABP1

NOTCH3

NUDT7

OGG1

PICALM

PIK3CA

PITX1

PLA2G2A

PMS1

PMS2

PMS2CL

POLD

POLD3

POLE

PPP1CB

PRADC1

PRSS37

PSPH

PTCHD3

PTEN

PTPRJ

RAI1

RHNP2

SFXN4

SHROOM2

SLC5A9

SMAD4

SMAD7

STK11

TBX3

TERC

TERT

TGFBR2

TKT

TLR2

TLR4

TP53

TRA2A

TREX2

TWSG1

UACA

UBAP2

USP6NL

ZFP14

ZMYM5

ZNF490
