# Supplementary material for: A novel POLE mutation associated with cancers of colon, pancreas, ovaries and small intestine
Source: Fam Cancer. 2015 Apr 10;14(3):437–48. doi: 10.1007/s10689-015-9803-2 (PMC4559173; doi:10.1007/s10689-015-9803-2)
Supplement: Supplementary file 2 — Supplementary material 2 (PDF 41 kb) [file 10689_2015_9803_MOESM2_ESM.pdf]

Online Resource 3: List of genes included in the endocrine tumour panel

Journal: Familial Cancer

Title: A Novel POLE Mutation Associated with Cancers of Colon, Pancreas, Ovaries and Small Intestine

Authors: Maren F. Hansen, Jostein Johansen, Inga Bjørnevoll, Anna E. Sylvander, Kristin S. Steinsbekk, Pål Sætrom, Arne K. Sandvik, Finn Drabløs, Wenche Sjursen.

Corresponding author:

Maren F. Hansen

Department of Laboratory Medicine, Children´s and Women´s Health, Faculty of Medicine, Norwegian University of Science and Technology, 7491 Trondheim, Norway.

Department of Pathology and Medical Genetics, St. Olavs Hospital, Trondheim University Hospital, 7006 Trondheim, Norway

maren.hansen@ntnu.no or maren.hansen@stolav.no

CDC73

CDKN2A

ALK

DICER1

BAP1

PALB2

FH

KIF1B

MAX

MEN1

MEN2

NF1

NF2

MET

PHOX2B

PTCH1

RAD51C

RET

RB1

SMARC1

TMEM127

TSC1

TSC2

VHL

SDHA

SDHAF2

SDHB

SDHC

SDHD
