# Supplementary material for: A novel POLE mutation associated with cancers of colon, pancreas, ovaries and small intestine
Source: Fam Cancer. 2015 Apr 10;14(3):437–48. doi: 10.1007/s10689-015-9803-2 (PMC4559173; doi:10.1007/s10689-015-9803-2)
Supplement: Supplementary file 3 — Supplementary material 3 (PDF 71 kb) [file 10689_2015_9803_MOESM3_ESM.pdf]

Online Resource 1: Table with overview of the sequenced individuals with regard to phenotype, *POLE* mutation carrier status, sequence methods used and which filtering strategy they were included in.

Journal: Familial Cancer

Title: A Novel POLE Mutation Associated with Cancers of Colon, Pancreas, Ovaries and Small Intestine

Authors: Maren F. Hansen, Jostein Johansen, Inga Bjørnevoll, Anna E. Sylvander, Kristin S. Steinsbekk, Pål Sætrom, Arne K. Sandvik, Finn Drabløs, Wenche Sjursen.

Corresponding author:

Maren F. Hansen

Department of Laboratory Medicine, Children´s and Women´s Health, Faculty of Medicine, Norwegian University of Science and Technology, 7491 Trondheim, Norway.

Department of Pathology and Medical Genetics, St. Olavs Hospital, Trondheim University Hospital, 7006 Trondheim, Norway

maren.hansen@ntnu.no or maren.hansen@stolav.no

ID	Phenotype	*POLE* p.Tyr458Phe carrier	Exome seq/avg coverage/ %covered target[1]	Validated by Sanger	Classified as affected/unaffected for filtering	Used in filtering based on disease status	Used in filtering based on CRC gene panel
III:2	CRC 63 y.	Yes	No/-/-	Yes	-	-	-
III:16	No cancers	No	Yes/97/96	Yes	Unaffected	Yes	Yes
IV:3	Lung cancer 72 y.	No	Yes/126/98	Yes	Unclassified	No	Yes
IV:6	Prostate cancer 59 y	No	No/-/-	Yes	-	-	-
IV:8	Pancreatic cancer 46 y.	Yes	No/-/-	Yes	-	-	-
IV:9	Bil. Ovarian cancer 40 y. CRC 48 y, Mult. adenomas	Yes	Yes/220/99	Yes	Affected	Yes	Yes
IV:10	Mult. adenomas	Yes	Yes/165/99	Yes	Affected	Yes	Yes
IV:12	Bladder cancer 54 y.	No	Yes/242/99	Yes	Unclassified	No	Yes
IV:13	CRC 43 y., Mult. adenomas	Yes	No/-/-	Yes	-	-	-
IV:15	CRC 58 y	Yes	No/-/-	Yes	-	-	-
IV:17	CRC 42 y, small intestine cancer 54 and 57 y., mult. adenomas	Yes	Yes/155/99	Yes	Affected	Yes	Yes
IV:20	CRC 38 y.	Yes	No/-/-	Yes	-	-	-
IV:21	CRC 56 y., adenomas	Yes	Yes/112/97	Yes	Unclassified	No	Yes
V:2	Adenoma	No	Yes/235/99	Yes	Unclassified	No	Yes
V:4	Mult. adenomas	Yes	Yes/157/98	Yes	Affected	Yes	Yes
V:5	Mult. adenomas	Yes	Yes/187/99	Yes	Affected	Yes	Yes
V:7	Rectal neuroendocrine tumour 34 y., adenomas.	No	Yes/117/98	Yes	Affected	Yes	Yes
V:8	Adenomas	Yes	Yes/81/95	Yes	Affected	Yes	Yes
V:9	-	No	Yes/159/99	Yes	Unclassified	Yes	Yes
V:10	-	No	Yes/68/93	Yes	Unclassified	Yes	Yes

[1]estimated as the percentage of target regions with an average coverage of at least 20x. The percentage of bases within target regions with a coverage of at least 20x is similar, between 91 and 99%.
